# Diagnostic performance of new and classic CSF biomarkers in age-related dementias

**DOI:** 10.18632/aging.101925

**Published:** 2019-04-27

**Authors:** Francesca Marchegiani, Giulia Matacchione, Deborah Ramini, Fiorella Marcheselli, Rina Recchioni, Tiziana Casoli, Elisa Mercuri, Marco Lazzarini, Belinda Giorgetti, Valentina Cameriere, Susy Paolini, Lucia Paciaroni, Tommaso Rossi, Roberta Galeazzi, Rosamaria Lisa, Anna Rita Bonfigli, Antonio Domenico Procopio, Maria De Luca, Giuseppe Pelliccioni, Fabiola Olivieri

**Affiliations:** 1 Center of Clinical Pathology and Innovative Therapy, IRCCS INRCA, Ancona, Italy; 2 Department of Clinical and Molecular Sciences, DISCLIMO, Università Politecnica delle Marche, Ancona, Italy; 3 Center for Neurobiology of Aging, IRCCS INRCA, Ancona, Italy; 4 Neurology Unit, IRCCS INRCA, Ancona, Italy; 5 Clinical Laboratory and Molecular Diagnostics, IRCCS INRCA, Ancona, Italy; 6 Scientific Direction, IRCCS INRCA, Ancona, Italy; 7 Department of Nutrition Sciences, University of Alabama at Birmingham, Birmingham, AL 35294, USA; * Equal contribution

**Keywords:** neurofilament-light, microRNAs, age-related dementias, CSF

## Abstract

The identification of diagnostic-prognostic biomarkers of dementia has become a global priority due to the prevalence of neurodegenerative diseases in aging populations. The objective of this study was to assess the diagnostic performance of cerebrospinal fluid (CSF) biomarkers across patients affected by either Alzheimer’s disease (AD), tauopathies other than AD (TP), or vascular dementia (VD), and cognitively normal subjects (CNS). One hundred fifty-three patients were recruited and tested for classical AD CSF biomarkers- Amyloid-ß42 and tau proteins - and novel candidate biomarkers - neurofilament (NF-) light and microRNA (miR) -21, -125b, -146a, and -222.

All dementia patients had significantly higher concentrations of NF-light compared to CNS, with the TP group displaying the highest NF-light values. A significant inverse correlation was also observed between NF-light and cognitive impairment. Of the four miRNAs analyzed, miR-222 levels were significantly increased in VD patients compared to both CNS and AD. In addition, while NF-light showed a better diagnostic performance than miR-222 and classical AD biomarkers in differentiating TP and VD from CNS, classical AD biomarkers revealed higher performance in discriminating AD from non-AD disorders.

Overall, our results suggest that CSF NF-light and miR-222 are promising biomarkers that may help to diagnose non-AD disorders.

## Introduction

The constant rate of increase in global life expectancy and the consequent rise in the average age of the population have been accompanied by a significant surge in the incidence of the most common age-related diseases (ARDs), including neurodegenerative diseases [1]. The economic costs and social burden associated with neurodegenerative diseases have motivated efforts to identify innovative biomarkers for accurate and timely diagnosis and effective treatments. Neurodegenerative diseases include Alzheimer's disease (AD), the most common form of dementia, and non-Alzheimer’s diseases (NAD), a group of disorders that account for approximately 30-40 per cent of dementias worldwide [2]. Among NAD, Lewy Bodies (DLB), vascular (VD) and frontotemporal dementia (FTD) are the most prevalent types of dementia.

AD diagnosis is currently based on clinical evaluation, neuropsychological testing, neuro-imaging techniques, and cerebrospinal fluid (CSF) classical biomarkers [3–6]. Three core CSF biomarkers, *e.g.* Amyloid-ß42 (Aß42), total tau (t-tau) and phosphorylated tau (p-tau) proteins, have been included in the diagnostic criteria of AD, and could be relevant for differential diagnosis [3]. A recent Cochrane review suggested that they have a better sensitivity than specificity, performing best in ruling out AD [5].

Tau is a microtubule-associated protein involved in microtubule assembly and stabilization that can form filamentous deposits that are hallmarks of several neurodegenerative diseases collectively referred to as tauopathies (TP). TPs include AD and non-AD diseases, such as FTD and progressive supranuclear palsy (PSP) [6].

The onset of clinical symptoms and signs is a late occurrence in the natural history of dementia since the neurodegenerative processes start decades before the characteristic clinical manifestations [7,8]. To date, there is no single test that can diagnose the different types of dementia and the identification of innovative diagnostic biomarkers that can contribute to distinguish AD from NAD is needed.

New molecules, such as neurofilament light (NF-light) and microRNAs, have been proposed as promising biomarkers for neurodegenerative diseases. Neurofilaments are the major cytoskeletal constituents of neuronal cells, involved in axonal caliber maintenance and morpho-functional integrity [8–10]. NF-light levels are correlated with axonal degeneration, suggesting a potential diagnostic relevance for AD [11,12]. Increased NF-light levels have been observed in a large number of neurodegenerative diseases and conditions, including multiple sclerosis (MS) [13,14], amyotrophic lateral sclerosis (ALS) [15], AD [12], subcortical vascular disease [16], FTD [17,18], various central nervous system infections [19], and chronic experimental autoimmune encephalomyelitis [20].

Circulating microRNAs (miRNAs), which are short single-strand RNA molecules that are involved in gene expression modulation, have been linked to a number of ARDs, including neurodegenerative diseases. Four miRNAs, miR-21, miR-125b, miR-146a, and miR-222, were previously associated with AD diagnosis [21–23]. Moreover, two of them, miR-21 and miR-146a, were found to be involved in the modulation of the inflammatory process, which in turn is currently believed to underlie the neurodegeneration processes [24]. These miRNAs were defined as “inflammamiRs” [25].

The aim of this study was to compare the diagnostic performance of classical and novel CSF biomarkers across patients affected by AD and NAD, such as TP and VD, and cognitively normal subjects (CNS).

## RESULTS

The biochemical, clinical and anthropometric characteristics of the studied subjects are reported in Table 1. The proportion between genders among groups was not significantly different. TP patients have mean age similar to that of CNS, whereas AD and VD patients were significantly older than CNS.

**Table 1:** Clinical and anthropometric characteristics of the studied subjects.

	***CNS*** ***(n. 43)***	***AD*** ***(n. 70)***	***TP*** ***(n. 23)***	***VD*** ***(n. 17)***
***Age (yrs)***	66.9±12.0	77.0±7.7*	68.6±8.3	79.4±6.2*
***Gender Male N (%)***	21 (48.8%)	26 (37.1%)	12 (52.2%)	8 (47.1%)
***Aß42*** ***(pg/ml)***	657.0 (143.0-1238.0)	384.5 (113.0-877.0) *	713.0 (194.0-1006.0)	645.0 (394.0-1107.0)
***T-tau (pg/ml)***	178.0 (57.0-2358.0)	447.0 (117.0-2693.0) *	247.0 (90.0-774.0)	305.0 (66.0-673.0)
***P-tau (pg/ml)***	35.0 (6.0-81.0)	64.0 (21.0-194.0) *	48 (2.0-112.0)	44.0 (25.0-91.0)
***MMSE***	25.3±3.1	14.9±6.3 *	18.2±7.7 *	20.3±7.8 *
***IATI***	1.4 (0.3-3.1)	0.5 (0.1-1.7) *	1.1 (0.3-2.1) *	1.3 (0.5-2.2)
***NF-light (pg/ml)***	796.7 (81.3-1584.3)	1332.7 (424.7-5730.1) **	2071.0 (400.5-7864.9) **	1603.2 (370.1-6295.7) ^++^
***MiR-21***	324.7±215.1	369.7±236.9	304.9±139.3	286.1±177.5
***MiR-125b***	171.8±165.5	166.6±151.2	129.1±122.1	167.0±199.9
***MiR-146a***	14.8±15.1	14.4±11.2	16.5±19.5	16.4±18.9
***MiR-222***	11.2±12.1	13.8±13.8	23. ±58.8	40.6±64.0^++^

Data are reported as mean ± SD or as median (Interquartile Range) as appropriate. Aß42: beta-amyloid (1-42) peptide. T-tau: Tau protein. P-tau: threonine-181 hyperphosphorylated tau protein. MMSE: Mini Mental State Evaluation. IATI: Innotest Amyloid Tau Index. CNS: cognitively normal subjects. AD: Alzheimer’s diseases. TP: the TP group was composed of 3 patients with progressive supranuclear palsy (PSP), 19 patients with frontotemporal dementia (FTD) and 3 patients with corticobasal degeneration (CBD). VD: vascular dementia. *p<0.05 vs. CNS group; **^++^** *p<0.01 vs.* CNS and AD group; ** *p< 0.01 vs.* CNS.

With respect to classical AD CSF biomarkers, AD patients showed the characteristic profiles characterized by low levels of Aß42 and high levels of t- and p-tau, whereas the CNS group had high levels of Aß42 and low levels of t- and p- tau. NAD patients showed intermediate profiles.

**Figure 1:**
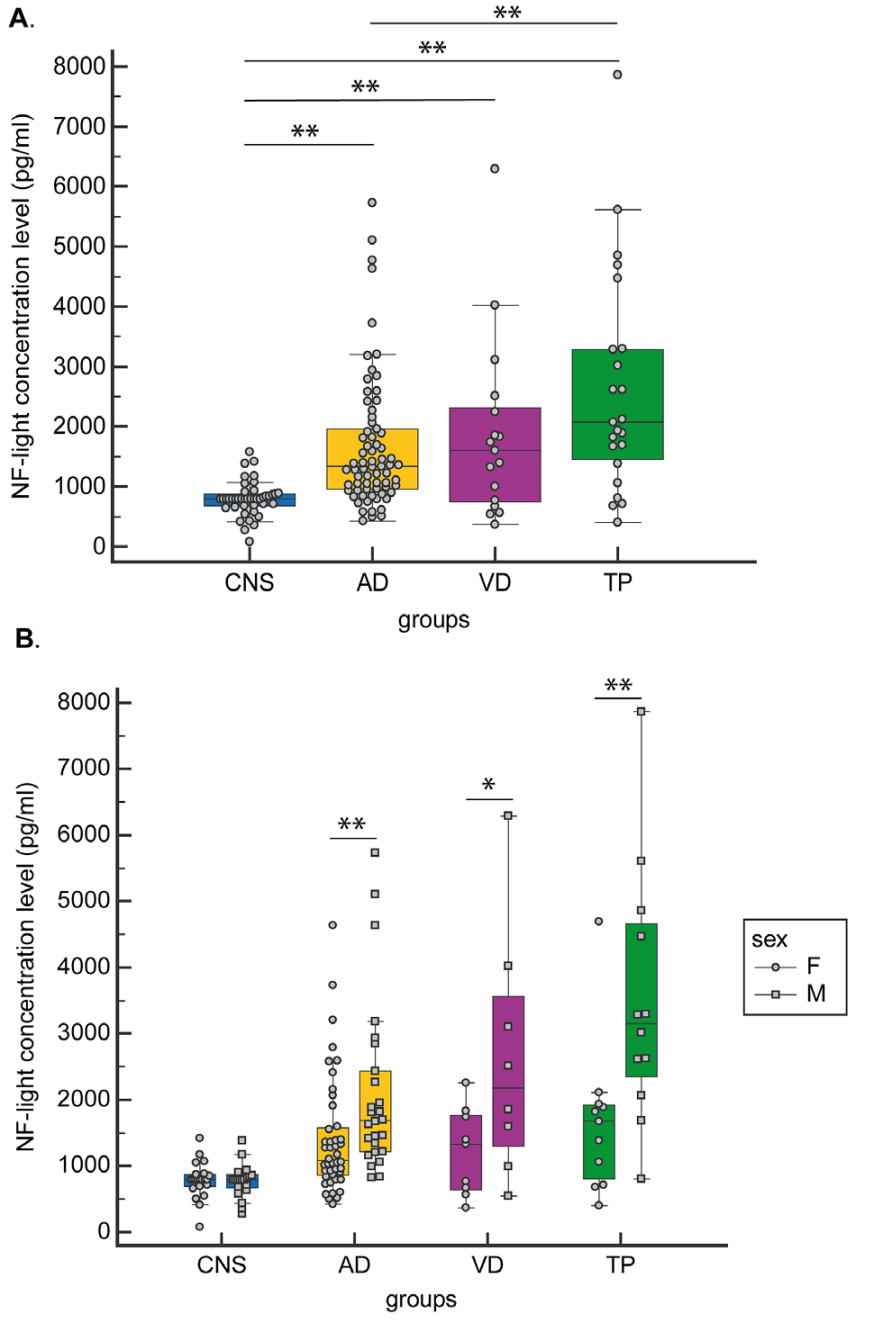
**CSF NF-light concentration levels.** (**A**) in CNS, AD, VD and TP and (**B**) in CNS, AD, VD and TP grouped by gender. Data are presented as median (Interquartile Range). *p<0.05; **p<0.01.

A significant increasing trend from CNS to AD and NAD was observed for NF-light concentration levels. Specifically, the TP group was characterized by the highest NF-light value (Table 1 and Fig. 1A).

When gender-stratified analyses were performed, NF-light levels were significantly higher in males than in females in both AD and NAD (TP and VD groups) (Fig. 1B).

**Figure 2:**
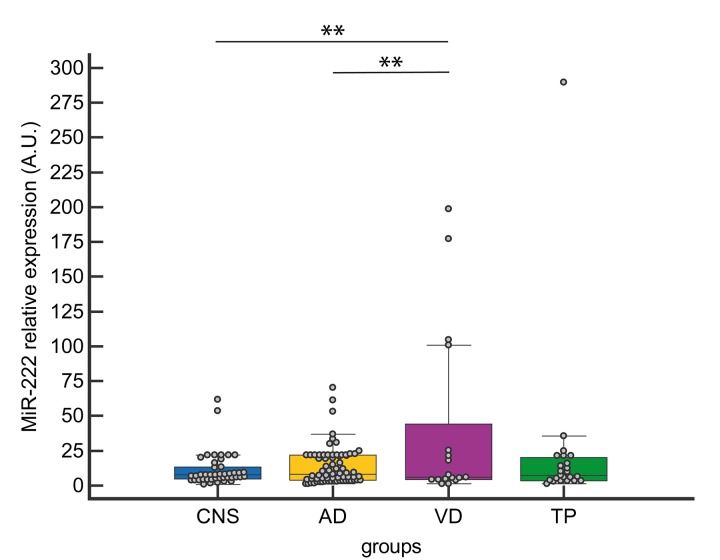
**CSF miR-222 expression levels in CNS, AD, VD and TP.** Data are presented as median [interquartile range]; **p<0.01

Among the four selected miRNAs, miR-222 CSF levels were significantly increased in VD compared to both AD and CNS (Fig. 2).

One-way analysis of covariance (ANCOVA) adjusted for age and gender confirmed a statistically significant difference among CNS, AD, TP and VD patients in the levels of NF-light (F =13.07 (3, 141), *p* <0.001) and miR-222 (F= 5.51 (3, 141), *p=*0.001), (Wilk’s Lambda, F= 9.20 (3, 141), *p* <0.001). These results were still significant after adjusting for Aß42, t-tau, and p- tau.

**Figure 3:**
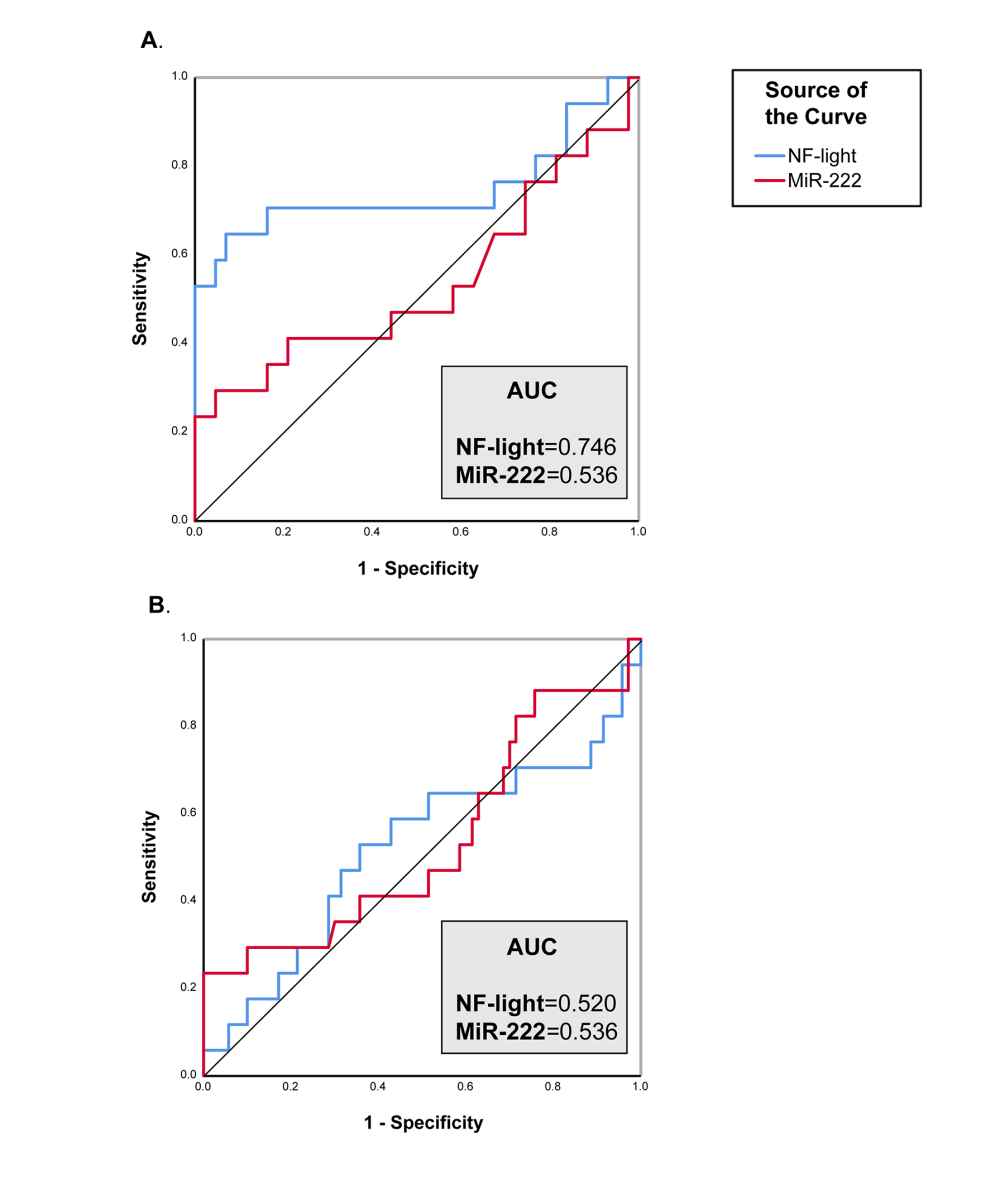
**ROC curve analysis of NF-Light and miR-222.** (**A**) CNS *vs.* VD. (**B**) AD *vs.* VD. AUC= Area Under the Curve.

The diagnostic performance of each biomarker was than assessed using ROC curve analysis. NF-light concentrations performed better than miR-222 levels in discriminating VD from CNS (ROC analysis: The diagnostic performance of each biomarker was than assessed using ROC curve analysis. NF-light concentrations performed better than miR-222 levels in discriminating VD from CNS (ROC analysis: AUC=0.746) (Fig. 3A), but neither NF-light nor miR-222 were able to distinguish between AD and VD (Fig. 3B).

**Figure 4:**
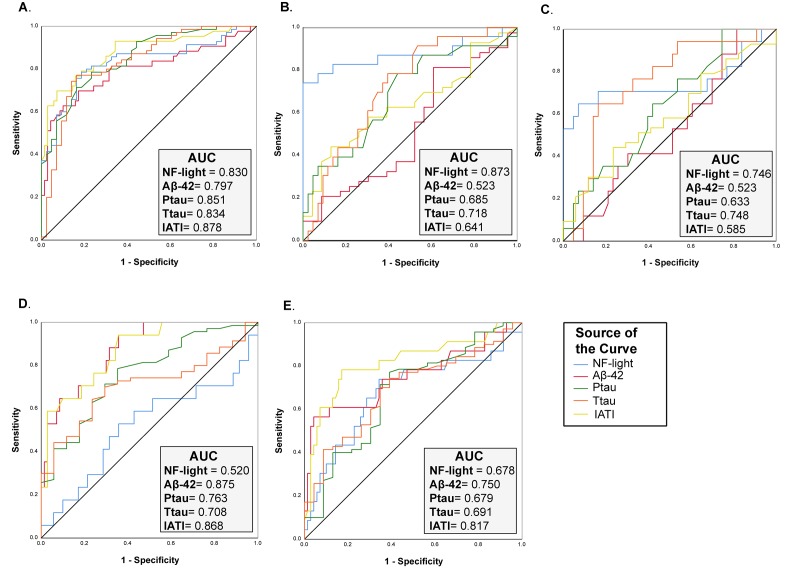
**ROC curve analysis of NF-Light and classical AD biomarkers.** (**A**) CNS *vs.* AD. (**B**) CNS *vs.* TP. (**C**) CNS *vs.* VD. (**D**) AD *vs.* VD and (**E**) AD *vs.* TP. AUC= Area Under the Curve.

On the other hand, the performance of NF-light levels in discriminating AD from CNS was similar to that of classical AD biomarkers (ROC analysis: AUC=0.830, AD *vs.* CNS) (Fig. 4A), whereas a better performance than Aß42, t- and p-tau and IATI (a parameter combining Aß42 and t-tau) was observed in differentiating TP patients from CNS (ROC analysis: AUC=0.873, TP *vs.* CNS) (Fig. 4B). Furthermore, IATI had the best performance in differentiating between AD and NAD (ROC analysis: AUC=0.868, AD *vs.* VD and AUC=0.817, AD *vs.* TP) (Fig. 4D-E).

Significant correlations were observed between NF-light and t-tau, p-tau and IATI (Table 2).

**Table 2:** Correlation between classical and innovative (NF-light and miR-222) neurodegenerative biomarkers.

	***NF-light***			***MiR-222***	
		***Spearman’s rho coefficient***	***P value***	***Spearman’s rho coefficient***	***P value***
***A*ß42**		*-0.031*	*0.692*	*-0.011*	*0.888*
***T-tau***		***0.360***	***< 0.001***	*-0.077*	*0.319*
***P-tau***		***0.262***	***0.001***	*-0.016*	*0.838*
***IATI***		***-0.210***	***0.006***	*0.040*	*0.603*
***MMSE***		***-0.210***	***0.022***	*0.026*	*0.786*

Aß42: beta-amyloid (1-42) peptide. T-tau: Tau protein. P-tau: threonine-181 hyperphosphorylated tau protein. MMSE: Mini Mental State Evaluation. IATI: Innotest Amyloid Tau Index.

Finally, a significant inverse correlation was observed between NF-light levels and the Mini-Mental State Examination (MMSE) score (Table 2).

## DISCUSSION

The confidence in AD diagnosis is highly enhanced by the quantification of classical biomarkers in CSF. However, the diagnostic relevance of these biomarkers in non-AD dementia and in differentiating between AD and non-AD is still under investigation. In addition, the identification of additional CSF biomarkers of dementia may help to improve the CSF biomarker-based classification. To this end, our results showed that CSF NF-light levels increased in all analyzed groups of patients with dementia compared to CNS. The TP group was characterized by the highest NF-light values, which, in turn, showed a better diagnostic performance than classical AD biomarkers in distinguishing TP from CNS. A notable finding of our study is that NF-light levels were strongly correlated with t-tau and p-tau, which confirms previous data [26]. This relationship is not surprising since t- and p-tau as well as NF-light are proteins belonging to the neuronal cytoskeleton and their increased levels in CSF are indicative of neuronal damages. The tau protein is mainly associated with the microtubules of the cortical axons; therefore, the value of t-tau in CSF is proportional to axonal damage. On the other hand, the axonal damage, in general, is an event common to all neurodegenerative diseases. Indeed, increases of t-tau are also detectable in VD, Lewy body dementia, FTD dementias and Creutzfeldt-Jacob disease [27,28]. Given that the tau protein is expressed mainly in the axons of cortical neurons, an increase in CSF reflects a neuronal damage of cortical neurons [29]. Nevertheless, the increase in CSF t-tau levels remains a finding rather non-specific, since other pathologies, besides AD, are responsible for most of the cortical neuronal damage, *i.e.* Creutzfeld's-Jacob disease [30]. NF-light as representative of axonal injury, axonal pathology, and/or axonal dysfunction has been observed in various models of mouse [26–31]. Moreover, results from this study showed a significant inverse correlation between NF-light and the MMSE score, the best-known screening tool for providing an overall measure of cognitive impairment in clinical and research field [32]. However, as seen previously [33], CSF NF-light levels demonstrated worse diagnostic performance in discriminating between AD and non-AD dementia, suggesting that NF-light is a biomarker of neurodegeneration not specific for AD dementia

On the other hand, IATI, a parameter combining Aß42 and t-tau that, had the best performance in discriminating between AD and non-AD dementia. This result is in contrast with findings from a recent Cochrane systematic review on the clinical impact of CSF t-tau and p-tau for the diagnosis of Alzheimer’s and other dementias in people with mild cognitive impairment (MCI), which reported that the classical AD biomarkers showed better potential in ruling out AD rather than in ruling in [5]. Despite the large number of patients analysed in the above-mentioned systematic review, the diagnostic clinical utility of these biomarkers was not defined. Indeed, an increase in their levels is not a guarantee of a progression of MCI into dementia, suggesting that further efforts should be devoted to search for more specific and informative biomarkers.

Regarding miRNAs levels as diagnostic biomarkers of dementia, our study showed a significant increase of CSF miR-222 levels in VD patients, suggesting a potential role in supporting the diagnosis of vascular dementia. MiR-222 was previously defined as an anti-angiogenic miR, upregulated in vascular walls with neointimal lesion formation [34–36]. Notably, circulating miR-222 levels were recently proposed as promising and independent biomarkers for the risk of acute ischemic stroke [37]. However, miR-222 performed worse than NF-light in distinguishing VD from CNS. The diagnostic relevance previously hypothesized for miR-21, miR-146a, and miR-125b CSF expression levels was not confirmed in our study [21–23]. Methodological bias, lack of standardization procedures, or different inclusion- exclusion criteria for enrolled patients could explain these non-concordant results.

The main limitation of our study is the small number of recruited patients. This is due to the fact that an invasive procedure is needed to collect CSF samples. Even if CSF biomarkers levels reflect the neuropathological alterations better than peripheral blood, applying routinely the invasive procedure needed for CSF collection in elderly patients can be difficult [38]. Therefore, the main effort in the future will be to measure NF-light in serum or plasma and compare the diagnostic performance of this innovative biomarker in CSF and in peripheral blood samples.

Overall, our results suggest that, although the non-specificity of NF-light CSF levels for the different types of dementia, CSF NF-light could be useful in supporting the diagnosis of non-AD dementia in combination with imaging and clinical data.

## MATERIALS AND METHODS

### Participants

One hundred fifty-three patients (67 males and 86 females) were consecutively admitted to the Neurology Unit of the Geriatric Hospital, IRCCS INRCA, of Ancona. The period of recruitment was from July 20, 2010 to July 17, 2017. Participants with CSF sample available were included in the study. The Institutional Review Board of INRCA approved the study protocol and all study participants, or their next of kin, provided written informed consent in the case of relevant cognitive impairment. All recruited subjects were between 38 and 90 years of age.

Based on the cognitive assessments and clinical diagnosis, the patients were grouped into the following diagnostic categories: 70 AD, 23 TP, 17 VD, and 43 defined as CNS. The TP group was composed of: 3 patients with PSP, 19 FTD, and 3 patients with corticobasal degeneration (CBD). Tauopathies and vascular dementia are classified as NAD.

All the participants underwent physical, neurological and neuropsychological assessments, including laboratory tests, brain imaging and the MMSE evaluation. MMSE values were recoded as: 24-30 = no cognitive impairment, 19-23= mild cognitive impairment, 10-18 = moderate cognitive impairment and < 9 = severe cognitive impairment.

The diagnosis of dementia was made according to consensus criteria (AD [3,39,40]; PSP [41]; FTD [42,43]; VD [44,45]; CBD [46]:).

CNS were enrolled by Neurology Unit of the Geriatric Hospital, IRCCS INRCA, of Ancona. They did not meet criteria for mild cognitive impairment [47] and did not have any signs of inflammatory or neurodegenerative disorders, or family history of neurodegenerative disease.

For the purpose of this study, patients with unidentified neurodegenerative disease or patients with different various diagnoses (*e.g.* psychiatric disorders, traumatic brain injury, alcoholism, metabolic encephalopathy) defined according to international criteria, were excluded.

### Cerebrospinal fluid collection and analysis

CSF was obtained by lumbar puncture in the L3/L4 or L4/L5 intervertebral space. CSF samples were collected in polypropylene vials and centrifuged at 2000 x g for 10 min to pellet residual cells and other insoluble material, then the supernatant was aliquoted and stored at -80 °C until use for biomarker determination. The CSF levels of Aß42, t-tau, and p-tau were determined using commercially available ELISA kits (Fujirebio Inc., Japan) according to the manufacturer’s instructions. Assay performance was monitored using internal and external quality control samples. All analyses were performed by the same investigators who were blinded to patients’ demographic, clinical, and cognitive data. The cutoff values of the CSF biomarkers considered as biochemical evidence of AD were determined in samples ran in the same laboratory as the CSF samples [48] and were Aß42 < 500 pg/ml, t-tau > 350 pg/ml, and p-tau > 50 pg/ml. The IATI parameter was calculated as follows: Aβ42 / (240 + 1.18 × t-tau).

The CSF NF-Light concentrations were measured using a commercial ELISA kit (IBL, Hamburg, Germany) as described by the manufacturer. All samples were ran in duplicate and the mean value was considered for the analysis. Samples with an intra-assay coefficient of variation below 10.0% were included in this study. The lower limit of quantification is 32 pg/ml and the upper limit is 10000 pg/ml.

### RNA isolation

Total RNA was isolated from CSF sample (100μl) using Total RNA Purification Kit (product #17200) by Norgen Biotek Corporation (Thorold, ON, Canada), according to the manufacturer’s specific recommendations. RNA was stored at − 80°C until use.

### Quantitative RT-PCR of mature miRNAs

MiRNA relative expression was measured as reported in [49].

### Statistical analysis

Baseline characteristics were determined using descriptive statistics. Mean ± standard deviation (SD) or median (interquartile range (IQR)) were reported for continuous variables. To assess the normal distribution of the data, a Kolmogorov-Smirnov test was performed and non-normally distributed data were log10 transformed. Absolute frequencies or percentages were described for categorical variables. ANCOVA followed by Bonferroni’s post-hoc test for multiple comparisons was used to compare the mean differences in clinical variables after adjustment for age and sex. Associations between the variables were tested using Spearman correlation test.

To assess the diagnostic performance of each biomarker in distinguishing CNS from AD and NAD, a receiver operating characteristic (ROC) curve analysis was performed, and areas under the curve (AUC) were compared.

All tests were two-sided, and significance was set at p< 0.05. Statistical analyses were performed using IBM SPSS (IBM Corp, Armonk, NY, USA) version 25.0.
